# The transcription factor POPEYE negatively regulates the expression of bHLH Ib genes to maintain iron homeostasis

**DOI:** 10.1093/jxb/erad057

**Published:** 2023-02-14

**Authors:** Meng Na Pu, Gang Liang

**Affiliations:** CAS Key Laboratory of Tropical Plant Resources and Sustainable Use, Xishuangbanna Tropical Botanical Garden, Kunming, Yunnan 650223, China; The College of Life Sciences, University of Chinese Academy of Sciences, Beijing 100049, China; CAS Key Laboratory of Tropical Plant Resources and Sustainable Use, Xishuangbanna Tropical Botanical Garden, Kunming, Yunnan 650223, China; The College of Life Sciences, University of Chinese Academy of Sciences, Beijing 100049, China; University College Dublin, Ireland

**Keywords:** bHLH Ib, bHLH IVc, Fe homeostasis, FER-LIKE IRON DEFICIENCY-INDUCED TRANSCRIPTION FACTOR (FIT), POPEYE

## Abstract

Iron (Fe) is an essential trace element for plants. When suffering from Fe deficiency, plants modulate the expression of Fe deficiency-responsive genes to promote Fe uptake. POPEYE (PYE) is a key bHLH (basic helix-loop-helix) transcription factor involved in Fe homeostasis. However, the molecular mechanism of PYE regulating the Fe deficiency response remains elusive in Arabidopsis. We found that the overexpression of *PYE* attenuates the expression of Fe deficiency-responsive genes. PYE directly represses the transcription of bHLH Ib genes (*bHLH38*, *bHLH39*, *bHLH100*, and *bHLH101*) by associating with their promoters. Although PYE contains an ethylene response factor-associated amphiphilic repression (EAR) motif, it does not interact with the transcriptional co-repressors TOPLESS/TOPLESS-RELATED (TPL/TPRs). Sub-cellular localization analysis indicated that PYE localizes in both the cytoplasm and nucleus. PYE contains a nuclear export signal (NES) which is required for the cytoplasmic localization of PYE. Mutation of the NES amplifies the repression function of PYE, resulting in down-regulation of Fe deficiency-responsive genes. Co-expression assays indicated that three bHLH IVc members (bHLH104, bHLH105/ILR3, and bHLH115) facilitate the nuclear accumulation of PYE. Conversely, PYE indirectly represses the transcription activation ability of bHLH IVc. Additionally, PYE directly negatively regulates its own transcription. This study provides new insights into the Fe deficiency response signalling pathway and enhances the understanding of PYE functions in Arabidopsis.

## Teaser

POPEYE (PYE) negatively regulates bHLH Ib genes via two different mechanisms, and the conditional nuclear localization of PYE is crucial to maintain Fe homeostasis in Arabidopsis.

## Introduction

Iron (Fe) is an essential trace element for all biological organisms. It participates not only in intracellular redox reactions, but also in the transmission of electrons. In plants, Fe participates in cellular respiration, photosynthesis and catalytic reactions of metal proteins (Kobayashi and Nishizawa, 2012). However, due to the high redox potential of Fe^3+^/Fe^2+^, the free Fe ions in the cell are prone to Fenton reaction, which activates reduced oxygen and produces harmful superoxide, causing damage to cells (Sheftel *et al.*, 2012; Dixon and Stockwell, 2014). Therefore, plants must maintain Fe homeostasis through a rigorous set of regulatory mechanisms.

Although Fe is abundant in the earth’s crust, it mostly exists in the form of Fe^3+^, and its solubility is extremely low in high pH and calcareous soils, which seriously affects efficiency of its utilization by plants. As soil salinization increases, Fe deficiency in plants becomes prevalent. In the long-term evolutionary process, plants have formed a set of sophisticated molecular mechanisms for Fe absorption (Römheld and Marschner, 1986; Kobayashi and Nishizawa, 2012; Grillet and Schmidt, 2019). Dicotyledonous plants and non-graminaceous monocotyledonous plants absorb Fe by a reduction-based strategy which consists of three components: H^+^-ATPase, Fe^3+^ reduction enzyme and Fe^2+^ transporter. In Arabidopsis, the H^+^-ATPase AHA2 secretes H^+^ to reduce soil pH and increase Fe solubility in the rhizosphere (Santi and Schmidt, 2009); the Fe^3+^ reductase FRO2 (FERRIC REDUCTION OXIDASE 2) converts Fe^3+^ to Fe^2+^ (Robinson *et al.*, 1999); and the Fe^2+^ transporter IRT1 (IRON-REGULATED TRANSPORTER 1) transports Fe^2+^ into root cells (Henriques *et al.*, 2002; Varotto *et al.*, 2002;  Vert *et al.*, 2002). Graminaceous plants utilize a chelation-based strategy, in which low molecular weight mugineic acids are secreted into the rhizosphere to bind Fe^3+^ and then the chelation complex is transported into plant roots (Roberts *et al.*, 2004).

When exposed to Fe deficiency conditions, plants activate their Fe uptake systems. A series of transcription factors have been characterized to regulate the Fe deficiency response in Arabidopsis and rice (Gao and Dubos, 2021; Riaz and Guerinot, 2021; Liang, 2022). FER-LIKE IRON DEFICIENCY-INDUCED TRANSCRIPTION FACTOR (FIT) is the master regulator of Arabidopsis strategy I-associated genes, such as *IRT1* and *FRO2*, since their expression is compromised in the *fit* loss-of-function mutant which cannot survive without extra Fe supplementation (Colangelo and Guerinot, 2004; Jakoby *et al.*, 2004; Yuan *et al.*, 2008; Schwarz and Bauer, 2020). However, FIT alone is not sufficient to induce the expression of *IRT1* and *FRO2*. Four bHLH Ib sub-group members (bHLH38, bHLH39, bHLH100, and bHLH101) interact with FIT, and the co-overexpression of FIT and bHLH Ib constitutively activate the expression of *IRT1* and *FRO2* (Yuan *et al.*, 2008; Wang *et al.*, 2013). Both *FIT* and bHLH Ib genes are inducible in response to Fe deficiency. Four bHLH IVc proteins, bHLH34/IRON DEFICIENCY TOLERANT1 (IDT1), bHLH104, bHLH105/IAA-LEUCINE RESISTANT3 (ILR3), and bHLH115, directly bind to the promoters of bHLH Ib genes and promote their expression (Zhang *et al.*, 2015; Li *et al.*, 2016; Liang *et al.*, 2017). As positive regulators of the Fe deficiency response, bHLH IVc members are not stimulated at the transcription level by Fe deficiency in Arabidopsis (Zhang *et al.*, 2015; Li *et al.*, 2016; Liang *et al.*, 2017). In fact, bHLH IVc proteins are regulated at the post-translation level, as BRUTUS (BTS), a candidate of Fe sensor, promotes their degradation (Selote *et al.*, 2015; Li *et al.*, 2021; Xing *et al.*, 2021). Despite the increase in BTS protein stability under Fe-deficient conditions, a class of small peptides, IRONMANs, inhibit the interactions between bHLH IVc (bHLH105/bHLH115) and BTS, resulting in the elevation of bHLH IVc proteins (Li *et al.*, 2021).

One member of the bHLH IVb sub-group, bHLH121/UPSTREAM REGULATOR of IRT1 (URI), also directly associates with the promoters of bHLH Ib genes and forms heterodimers with bHLH IVc members to activate the expression of bHLH Ib genes (Kim *et al.*, 2019; Gao *et al.*, 2020; Lei *et al.*, 2020). The induction of *FIT* is blocked in the *bhlh121*/*uri* loss-of-function mutants (Kim *et al.*, 2019; Gao *et al.*, 2020; Lei *et al.*, 2020), and the *FIT* promoter is also bound by bHLH121 (Lei *et al.*, 2020). In addition to bHLH121, the bHLH IVb sub-group also contains bHLH11 and POPEYE (PYE/bHLH47). bHLH11 is a negative regulator containing two EAR motifs which recruit the transcriptional co-repressors TOPLESS/TOPLESS-RELATED (TPL/TPRs; Tanabe *et al.*, 2019; Y. Li *et al.*, 2022). Moreover, bHLH11 interacts with bHLH IVc proteins and interferes with their transactivation of bHLH Ib genes (Y. Li *et al.*, 2022). In contrast, PYE negatively regulates the expression of *NICOTIANAMINE SYNTHASE 4* (*NAS4*), *FRO3*, and *ZINC-INDUCED FACILITATOR1* (*ZIF1*) by directly binding to their promoters (Long *et al.*, 2010). Although PYE represses these Fe deficiency-inducible genes, its loss-of-function mutant displays the enhanced sensitivity to Fe deficiency. Muhammad *et al.*, (2022) reported that intercellular localization of PYE mediates cell-specific Fe deficiency responses. Similar to other bHLH Ib members, PYE interacts with three bHLH IVc members (bHLH104, bHLH105, and bHLH115) in Arabidopsis (Long *et al.*, 2010; Selote *et al.*, 2015; Tissot *et al.*, 2019); however, the biological significance of their protein interactions is still unclear. In the present study, we show that the cytoplasmic localization of PYE is required for the maintenance of Fe homeostasis in Arabidopsis. PYE represses the expression of bHLH Ib genes both directly and indirectly. Moreover, the transcription of *PYE* is also under the control of PYE itself.

## Materials and methods

### Plant materials and growth conditions

*Arabidopsis thaliana* ecotype Columbia-0 was used as the wild type in this study. *pye-1* was described previously (Long *et al.*, 2010). Surface-sterilized seeds were stratified at 4 °C for 1 d before being planted on medium. The Fe-sufficient medium used was half-strength Murashige and Skoog (MS) medium with 1% (w/v) sucrose, 0.7% (w/v) agar A, and 0.1 mM FeEDTA. Fe-deficient medium was the same, without FeEDTA. Arabidopsis and *Nicotiana benthamiana* were grown at 22 °C (14 h daylength, light intensity of 120 μmol m^–2^ s^–1^).

### Plasmid construction

Standard molecular biology techniques were used for the cloning procedures. Genomic DNA from Arabidopsis was used as the template for amplification of the upstream regulatory promoter sequences of *PYE*, *bHLH38*, and *bHLH39*. For *Pro*_*PYE*_:*HA*-*PYE-GFP*, *Pro*_*PYE*_:*HA*-*PYE*^*mEAR*^*-GFP* and *Pro*_*PYE*_:*HA*-*PYE*^*mNES*^*-GFP*, various versions of *PYE* sequences were inserted between the *PYE* promoter and poly(A) of the binary vector pOCA28. For overexpression of various versions of *PYE*, the HA tagged *PYE* (or *PYE*^*mEAR*^ or *PYE*^*mNES*^) was inserted between the CaMV 35S promoter and poly(A) of the binary vector pOCA30. Primers used for construction of these vectors are listed in Supplementary Table S1. Arabidopsis transformation was conducted by the floral dip method (Clough and Bent, 1998). Transgenic plants were selected with 50 μg ml^–1^ kanamycin.

### Fe reductase activity

Ferric chelate reductase assays were performed as described previously (Yi and Guerinot, 1996). Briefly, 10 intact plants for each genotype were pre-treated for 30 min in plastic vessels with 4 ml of half-strength MS solution without micronutrients (pH 5.5), and then soaked in 4 ml of Fe (III) reduction assay solution [half-strength MS solution without micronutrients, 0.1 mM Fe (III)-EDTA, and 0.3 mM ferrozine, pH adjusted to 5 with KOH for 30 min in darkness]. An identical assay solution containing no plants was used as a blank. The purple-coloured Fe (II)–Ferrozine complex was quantified at 562 nm.

### Gene expression analysis

One microgram of total RNA was used for oligo(dT)_18_-primed cDNA synthesis according to the reverse transcription protocol (TaKaRa, Japan). The resulting cDNA was subjected to relative quantitative PCR using the SYBR Premix Ex Taq kit (TaKaRa, Japan) on a Roche LightCycler 480 real-time PCR machine (Roche, Switzerland), according to the manufacturer’s instructions. For the quantification of each gene, at least three biological repeats were used. Gene copy number was normalized to that of *ACT2* (*ACTIN2*) and *PP2A* (* PROTEIN PHOSPHATASE 2A*). Primers used for qRT–PCR were described previously (Lei *et al.*, 2020; Y. Li *et al.*, 2022).

### Sub-cellular localization

GFP and mCherry were cloned into pOCA30 to generate *Pro*_*35S*_*:GFP* and *Pro*_*35S*_*:mCherry*, respectively. PYE was fused with mCherry in *Pro*_*35S*_*:mCherry* to generate *Pro*_*35S*_*:mCherry-PYE*. The coding sequences of *bHLH34*, *bHLH104*, *bHLH105*, and *bHLH115* were amplified from Arabidopsis root cDNA and cloned into *Pro*_*35S*_*:GFP* to generate *Pro*_*35S*_*:bHLH34-GFP*, *Pro*_*35S*_*:bHLH104-GFP*, *Pro*_*35S*_*:bHLH105-GFP*, and *Pro*_*35S*_*:bHLH115-GFP*, respectively (Lei *et al.*, 2020). *Pro*_*35S*_*:mCherry-PYE* was co-expressed with various GFP-containing vectors in 3-week-old *N. benthamiana* epidermal cells. Epidermal cells were observed under an Olympus confocal microscope (OLYMPUS, Japan). Excitation laser wavelengths of 488 nm and 563 nm were used for imaging GFP and mCherry signals, respectively.

### Determination of fluorescence ratio

Total intensity of the fluorescence from the nucleus and cytoplasm was measured separately by ImageJ (Version 1.52a). The ratio was calculated for each individual cell. Ten cells were processed per fluorescent reporter under each condition. Two independent experiments were conducted with similar results.

### Transient expression assays in *Nicotiana benthamiana*

*Agrobacterium tumefaciens* strain EHA105 was used in the transient expression experiments in *N. benthamiana*. Agrobacterial cells were infiltrated into leaves of *N. benthamiana* by the infiltration buffer (0.2 mM acetosyringone, 10 mM MgCl_2_ and 10 mM MES, pH 5.6). In the transient expression assays, the final OD_600_ value was 1. After infiltration and incubation of plants for 2 d in the dark, GFP fluorescence was observed through a confocal laser scanning microscope, and leaf samples were harvested. *pGAL4* promoter and BD domain were described previously (Li *et al.*, 2016). nGFP (or nmCherry) was generated by fusing the SV40 NLS with nGFP (or nmCherry). *pGAL4* promoter was fused with nGFP and cloned into the pOCA28 binary vector. For the generation of *Pro*_*35S*_*:BD-nmCherry*, the GAL4 BD was fused with nmCherry and cloned downstream of the 35S promoter in the pOCA30 binary vector. bHLH105 (or bHLH115) was fused to the C terminus-end of mCherry in *Pro*_*35S*_*:BD-nmCherry*. For *Pro*_*35S*_*:HA-PYE*, HA tagged PYE was cloned downstream of the 35S promoter in the pOCA30 vector. For co-infiltration, various agrobacterial cells were mixed prior to infiltration. Leaf infiltration was conducted in 3-week-old *N. benthamiana*. *NPTII* gene in the pOCA28 vector was used as the internal control. *GFP* transcript abundance was normalized to that of *NPTII*.

### Transient expression assays in Arabidopsis protoplasts

Arabidopsis protoplast preparation and subsequent transfection were performed as described previously (Wu *et al.*, 2009). GFP and PYE-GFP were transfected separately into protoplasts. After incubation for 12 h, the protoplasts were harvested and fluorescence emission of GFP in protoplasts was observed.

### Yeast-two-hybrid assays

Yeast transformation was performed according to the Yeastmaker Yeast Transformation System 2 User Manual (Clontech). Growth was determined as described in the yeast two-hybrid system user manual (Clontech).

### Immunoblotting

For total protein isolation, samples were ground in liquid nitrogen and resuspended in RIPA buffer (50 mM Tris, 150 mM NaCl, 1% NP-40, 0.5% sodium deoxycholate, 0.1% SDS, 1 mM PMSF, 1 × protease inhibitor cocktail, pH 8.0). Nuclear and cytosolic proteins were extracted as described previously (Saleh *et al.*, 2008) with minor modifications. In brief, Arabidopsis tissues were ground and resuspended in 2 ml of pre-chilled nuclei isolation buffer (0.25 M sucrose, 15 mM PIPES, pH 6.8, 5 mM MgCl_2_, 60 mM KCl, 15 mM NaCl, 1 mM CaCl_2_, 0.9% Triton X-100, and 1× protease inhibitor cocktail, Roche). After homogenization, the slurry was filtered and centrifuged at 12 000 ×*g* for 20 min at 4 °C. The proteins from the supernatant were extracted as cytosolic proteins, whereas the pellet resuspended in cold nuclei lysis buffer (50 mM HEPES, pH 7.5, 150 mM NaCl, 1 mM EDTA, 1% SDS, 0.1% Na deoxycholate, 1% Triton X-100, and 1× protease inhibitor cocktail, Roche) was collected as nuclear proteins. Samples were loaded onto 12% SDS–PAGE gels and transferred to nitrocellulose membranes. The membrane was blocked with Tris buffer saline with Tween-20 [TBST; 10 mM Tris–Cl, 150 mM NaCl, and 0.05% (v/v) Tween-20, pH 8.0] containing 5% (w/v) non-fat milk (TBSTM) at 22 °C for 60 min, and incubated with a primary antibody in TBSTM overnight at 4 °C. Membranes were washed with TBST (three times for 5 min each) and then incubated with the appropriate horseradish peroxidase conjugated secondary antibodies in TBSTM at 22 °C for 1.5 h. After washing three times, bound antibodies were visualized with enhanced chemiluminescence (ECL) substrate. The antibodies used for western blotting were as follows, mouse monoclonal anti-GFP (Abmart, China, 1:5000), anti-mCherry (Abmart, China, 1:5000), anti-Histone3 (Abmart, China, 1:5000), and goat anti-mouse IgG horseradish peroxidase (Affinity Biosciences, America, 1:10 000).

### Electrophoretic Mobility Shift Assay (EMSA)

EMSA was conducted using the Chemiluminescent EMSA Kit (Beyotime, China) following the manufacturer’s protocol. The coding sequence of PYE was amplified from Arabidopsis root cDNA and cloned into pGEX-4T-1 vector. The recombinant GST-PYE protein was expressed and purified from *E. coli*. The culture solution was incubated with 0.5 M isopropyl β-D-1-thiogalactopyranoside at 16 °C for 16 h, and protein was extracted and purified by using the GST-tag Protein Purification Kit (Beyotime, China) following the manufacturer’s protocol. The DNA fragments with E-box (CANNTG) were used as probes. The promoter probes of *bHLH38* and *bHLH39* were described previously (Lei *et al.*, 2020). A DNA fragment of *PYE* promoter, which is bound by bHLH104 and bHLH105 (Zhang *et al.*, 2015), was used as the *PYE* promoter probe. Two complementary single-strand DNA primers with the 5’ terminus labelled with biotin were synthesized and annealed to form double-stranded DNA as probes. The annealing reaction solution for 1 × probe was as follows: 1 μl of 10 μM forward prime, 1 μl of 10 μM reverse primer, 3 μl of 10 × Taq buffer, and 25 μl of H_2_O. The annealing reaction solution for 100 × probe was as follow: 10 μl of 100 μM forward primer, 10 μl of 100 μM reverse primer, 3 μl of 10 × Taq buffer, and 7 μl of H_2_O. Reaction solution was incubated at 95 °C for 2 min, and cooled at 22 °C. The binding reaction solution was as follows: 5 μl of H_2_O, 2 μl of 5 × EMSA/Gel-Shift binding buffer, 2 μl of protein, 1 μl of labelled probe. The competitive binding reaction solution was as follows: 4 μl of H_2_O, 2 μl of 5 × EMSA/Gel-Shift binding buffer, 2 μl of protein (1 μg), 1 μl of 1 × labelled probe, and 1 μl of 100 × unlabelled probe. The binding reactions were incubated at 22 °C for 20 min. Unlabelled or E-box mutated DNA probes were synthesized and used as competitors, and the GST protein alone was used as the negative control. The single-strand DNA sequences are listed in Supplementary Table S1.

## Results

### Overexpression of *PYE* suppresses both FIT-dependent and FIT-independent Fe deficiency response

**Fig. 1.**
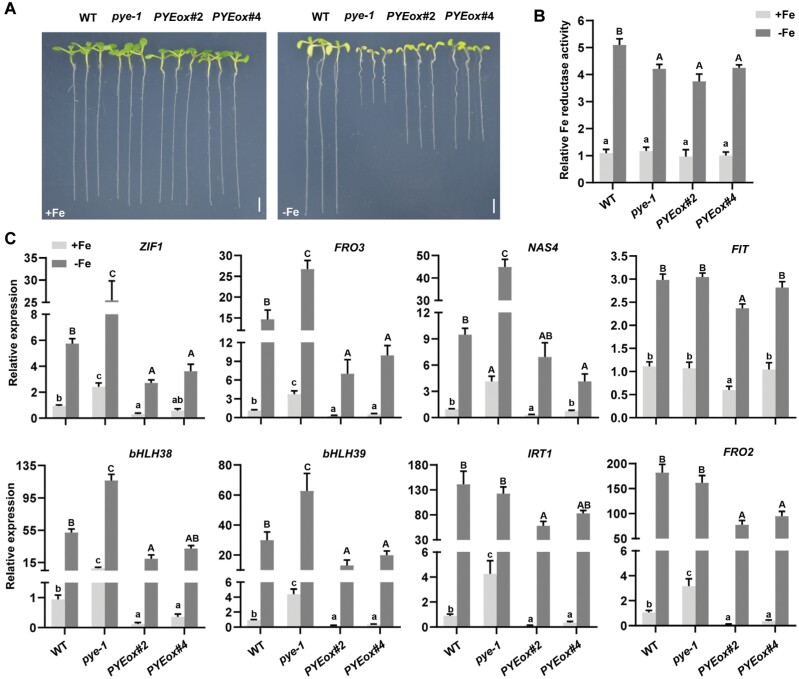
Characterization of *PYE* overexpressing plants. (A) Phenotypes of *PYEox* and *pye-1* plants. Seedlings were germinated and grown on Fe sufficient (+Fe) or Fe deficient (–Fe) medium for a week. Scale bars=4 mm. (B) Fe reductase activity. Four-day-old seedlings grown on +Fe medium were transferred to +Fe or –Fe medium for 3 d. The ferrozine assay was performed in triplicate, on 10 pooled plant roots. (C) Expression of Fe deficiency-responsive genes, *ZIF1*, *FRO3*, *NAS4*, *FIT*, *bHLH38*, *bHLH39*, *IRT1*, and *FRO2*. Four-day-old plants grown on +Fe medium were transferred to +Fe or –Fe medium for 3 d, and root samples were used for RT–qPCR. The expression levels were normalized to *ACT2* and *PP2A*. For (B) and (C) the data represent means ±SD. Different letters (lower case for +Fe, and upper case for –Fe) above each bar indicate statistically significant differences as determined by one-way ANOVA followed by Tukey’s multiple comparison test (*P*<0.05).

To further investigate the functions of PYE in the Fe deficiency response, we constructed *PYE* overexpressing plants (*PYEox*), in which HA-tagged *PYE* was driven by the CaMV 35S promoter (Supplementary Fig. S1). Under Fe-sufficient conditions, no visible difference was observed between *PYEox* and wild type plants. In contrast, under Fe-deficient conditions, similar to *pye-1*, the *PYEox* plants displayed sensitivity to Fe deficiency, such as short roots (Fig. 1A). We then analysed their Fe reductase activity (Yi and Guerinot, 1996). The results indicated that the Fe reductase activity was lower in the *PYEox* plants than in the wild type under Fe-deficient conditions (Fig. 1B). These data suggest that *PYE* overexpression disrupts the Fe deficiency response.

Next, we investigated whether the transcription of Fe deficiency response genes is affected in the *PYEox* plants. qRT–PCR was used to examine the expression of genes involved in the Fe deficiency response, including *ZIF1*, *FRO3*, *NAS4*, *FIT*, *bHLH38*, *bHLH39*, *IRT1*, and *FRO2* (Fig. 1C). In agreement with a previous study (Long *et al.*, 2010), the expression of *FIT*, *IRT1*, and *FRO2* was not significantly changed in the *pye-1* compared with the wild type. In contrast, all the above-mentioned genes were significantly down-regulated (*P*<0.05) in the *PYE*ox plants irrespective of the Fe status. These results suggest that the overexpression of *PYE* represses not only the FIT-independent genes, but also the FIT-dependent genes.

### PYE directly regulates the expression of bHLH Ib genes

**Fig. 2.**
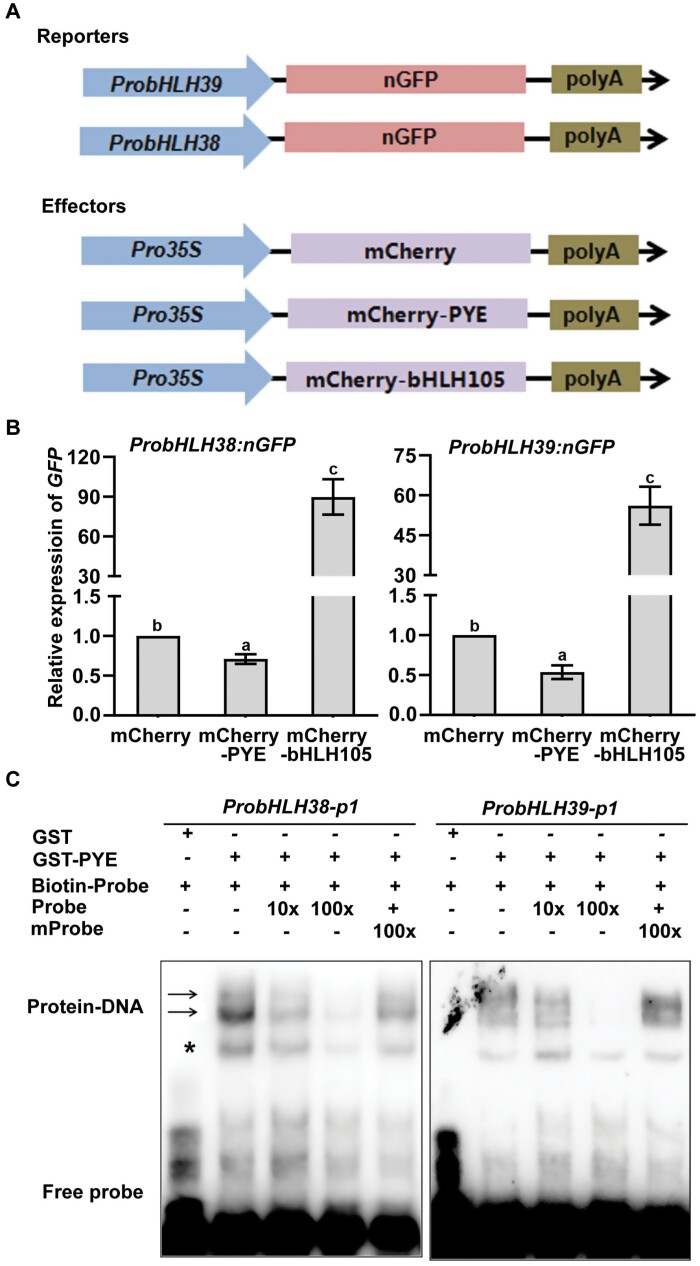
PYE directly regulates bHLH Ib genes. (A) Schematic representation of the constructs used for transient expression assays. The promoters of *bHLH38* and *bHLH3*9 were fused with a nucleus localized GFP (nGFP) as the reporters. The mCherry tag was fused with *PYE* driven by the CaMV 35S promoter, and used as an effector. *Pro*_*35S*_*:mCherry-bHLH105* and *Pro*_*35S*_*:mCherry* were used as the positive and negative controls, respectively. (B) *GFP* transcript abundance. *Agrobacterium* cells with the reporter or the effector were mixed and infiltrated into 3-week-old *Nicotiana benthamiana* leaves, and then kept in the dark for 48 h. The infiltrated leaves were harvested for RNA extraction and qRT–PCR. The abundance of *GFP* was normalized to that of *NPTII*. The value with the empty vector as an effector was set to 1. Each bar represents the mean ±SD of three independent experiments. Different letters above each bar indicate statistically significant differences as determined by one-way ANOVA followed by Tukey’s multiple comparison test (*P*<0.05). (C) EMSA showing that PYE directly binds to the *bHLH38* and *bHLH39* promoters. Either GST-PYE or GST was incubated with the biotin-labelled probes. Biotin-Probe, biotin-labelled probe; Probe, unlabelled probe; mProbe, unlabelled probe with mutated E-box. Biotin probe incubated with GST served as the negative control. Arrows indicate the GST-PYE/DNA complex; Asterisk indicates non-specific bands.

*PYE* overexpression significantly represses the expression of bHLH Ib genes, whereas the loss-of-function of *PYE* causes the opposite result (Fig. 1C), indicating that PYE negatively regulates bHLH Ib genes. Four bHLH IVc proteins are the positive regulators of bHLH Ib genes, and three of them physically interact with PYE (Long *et al.*, 2010). It is possible that PYE inhibits the activation of bHLH IVc proteins to regulate bHLH Ib genes. However, we cannot exclude the possibility that PYE alone directly regulates bHLH Ib gene expression. To verify this hypothesis, we performed transient expression assays (Fig. 2A). The promoters of *bHLH38* and *bHLH39* were fused with a nucleus localized GFP (nGFP) as the reporter, to yield *Pro*_*bHLH38*_*:nGFP* and *Pro*_*bHLH39*_*:nGFP.* The mCherry tag was fused with PYE and driven by the CaMV 35S promoter as an effector, to yield *Pro*_*35S*_*:mCherry-PYE*. *Pro*_*35S*_*:mCherry-bHLH105* and *Pro*_*35S*_*:mCherry* were used as the positive and negative controls, respectively. Each reporter was co-expressed with each of the effectors respectively. Compared with mCherry, mCherry-PYE significantly inhibited the expression of *GFP*, whereas mCherry-bHLH105 considerably promoted its expression (Fig. 2B). These results suggest that PYE alone is sufficient to repress expression of bHLH Ib genes.

It has been confirmed that bHLH IVc and bHLH121/URI directly bind to the promoters of bHLH Ib genes (Zhang *et al.*, 2015; Li *et al.*, 2016; Liang *et al.*, 2017; Kim *et al.*, 2019; Gao *et al.*, 2020; Lei *et al.*, 2020). Since both bHLH121 and PYE belong to the bHLH IVb sub-group (Heim *et al.*, 2003), we speculated that PYE directly regulates the transcription of bHLH Ib genes. To this aim, we conducted EMSAs (Fig. 2C). The promoters of *bHLH38* and *bHLH39* were used as the probes. The GST tagged PYE recombinant protein was expressed and purified in *Escherichia coli* (Supplementary Fig. S2). The biotin labelled probes were incubated with GST-PYE and the shifted DNA-protein complexes were detected. When excessive probes without biotin were added, the number of DNA-protein complexes decreased dramatically. In contrast, the addition of mutant probes did not cause the reduction of DNA-protein complexes. As the negative control, GST protein alone could not bind to the promoters of *bHLH38* and *bHLH39* (Fig. 2C). Taken together, these data suggest that PYE directly represses the transcription of *bHLH38* and *bHLH39* by association with their promoters.

### PYE has a non-functional EAR motif

**Fig. 3.**
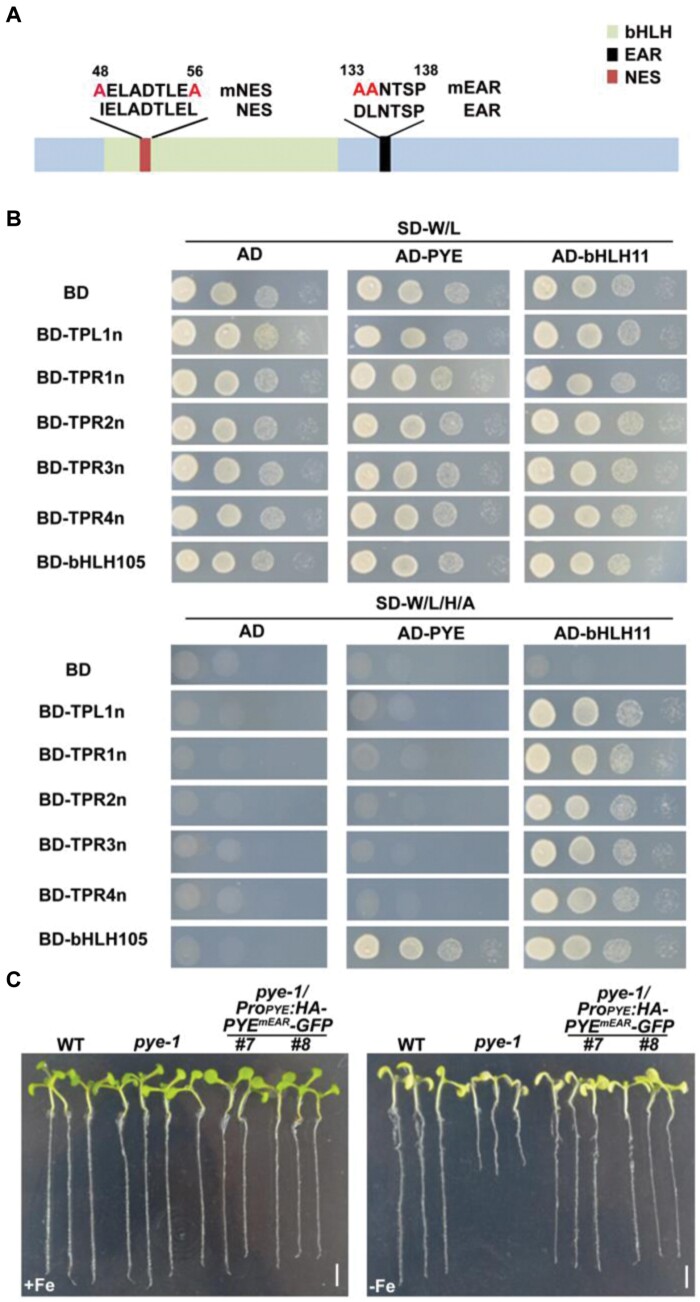
The repression function of PYE is independent of the EAR motif. (A) Schematic diagram of the various mutated versions of PYE. The mutated amino acids are indicated in red. mNES, the mutated NES. mEAR, the mutated EAR. (B) Yeast two-hybrid assays. Yeast co-transformed with different BD and AD plasmid combinations was spotted in parallel in a 10-fold dilution series. Growth on selective plates lacking Leu/Trp (SD–W/L) or Trp/Leu/His/Ade (SD–W/L/H/A). bHLH11 was used as the positive control for TPL/TPRs, and bHLH105 for PYE. (C) Complementation of the *pye-1* mutant by *PYE*^*mEAR*^. *PYE*^*mEAR*^ was fused with HA and GFP and driven by the *PYE* promoter. One-week-old seedlings grown on +Fe or –Fe medium. Scale bars=4 mm.

Having confirmed that PYE negatively affects the expression of Fe homeostasis-associated genes, we questioned how PYE might exert its negative regulatory function. It has been hypothesized that PYE might recruit the co-transcription repressors, TPL/TPRs (Szemenyei *et al.*, 2008; Pauwels *et al.*, 2010; ­Causier *et al.*, 2012), since it has an EAR motif (DLNxxP) in its C-terminal region (Fig. 3A). To confirm this hypothesis, we employed yeast-two-hybrid assays to test their protein interactions (Fig. 3B). The N-terminal regions of TPL/TPRs fused with BD (Binding Domain of GAL4 protein) were used as the baits, and PYE with AD (Activation Domain of GAL4 protein) as the prey. bHLH11 with AD was used as a positive control (Y. Li *et al.*, 2022). Yeast growth indicated that PYE cannot interact with all TPL/TPRs (Fig. 3B).

To further investigate whether the EAR motif is responsible for the negative regulation of PYE, we generated a mutated version of PYE (PYE^mEAR^, a PYE version containing a mutated EAR motif). *PYE*^*mEAR*^ was fused with HA and GFP and driven by the *PYE* promoter. The *Pro*_*PYE*_*:HA-PYE*^*mEAR*^*-GFP* construct was introduced into the *pye-1* mutant plants. Regardless of Fe status the *pye-1* mutant was completely rescued (Fig. 3C).

To further verify whether the EAR motif is required for the repression function of PYE, we constructed the *PYE*^*mEAR*^ overexpressing plants (*PYE*^*mEAR*^*ox*), in which HA tagged *PYE*^*mEAR*^ was driven by the CaMV 35S promoter (Supplementary Fig. S3A, B). Under Fe-deficient conditions, the Fe deficiency symptoms in the *PYE*^*mEAR*^*ox* plants were as severe as those in the *PYEox* plants (Supplementary Fig. S3C). We also added an LxLxL type of EAR motif to the C-terminus end of PYE and generated the *PYE*^*EAR*^*ox* plants (Supplementary Fig. S3A, D). There were no visible differences between the *PYE*^*EAR*^*ox* and *PYEox* plants (Supplementary Fig. S3C). Taken together, our data suggest that PYE cannot recruit the TPL/TPRs co-repressors, and its EAR motif is not required for its repressive function.

### PYE contains a nuclear export signal (NES) which is required for its functions

**Fig. 4.**
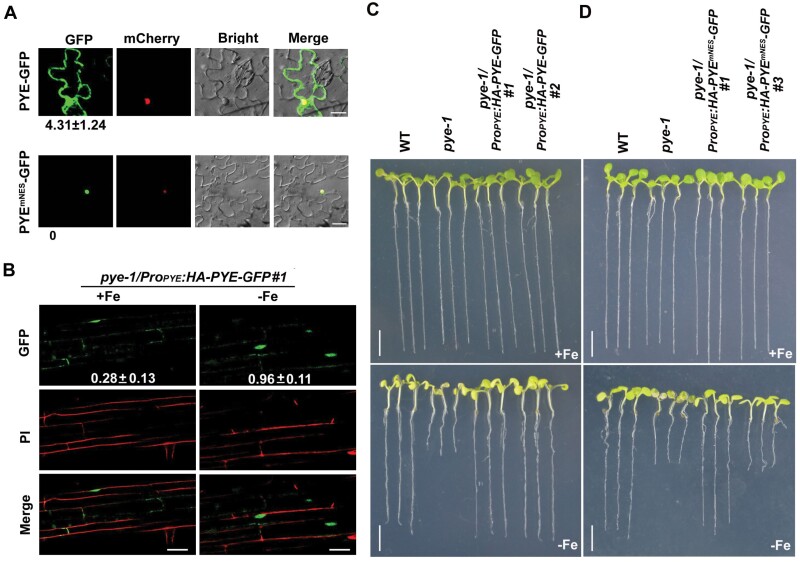
The NES is required for PYE functions. (A) Sub-cellular localization of PYE. PYE-GFP or PYE^mNES^-GFP was co-expressed with a NLS (nuclear localization signal) fused to mCherry in tobacco cells. The fluorescence signal was visualized under a confocal microscope. The numbers indicate the cytoplasm-to-nucleus ratio of fluorescence. The data represent means ±SD (*n*=10). Scale bars =20 μm. (B) Sub-cellular localization of PYE in response to Fe deficiency. One-week-old *pye-1/Pro*_*PYE*_*:HA-PYE-GFP* seedlings were grown on +Fe or –Fe medium. Roots were stained with propidium iodide (PI, in red). Root maturation regions are shown. Scale bars=25 μm. The numbers indicate the nucleus-to-cytoplasm ratio of fluorescence. (C) Phenotypes of *pye-1/Pro*_*PYE*_*:HA-PYE-GFP* plants. One-week-old seedlings grown on +Fe or –Fe medium. Scale bars=4 mm. (D) Phenotypes of *pye-1/Pro*_*PYE*_*:HA-PYE*^*mNES*^*-GFP* plants. One-week-old seedlings grown on +Fe medium. Scale bars=4 mm.

In the transient expression assays (Fig. 2A, B), we observed that the mCherry-PYE proteins exist in both the cytoplasm and nucleus. To validate this observation, we generated the PYE-GFP construct and performed transient expression assays in tobacco leaves and Arabidopsis protoplasts. The results indicated that PYE-GFP fusion proteins were also localized in the cytoplasm and nucleus (Fig. 4A; Supplementary Fig. S4A). We also generated and examined the *pye-1/Pro*_*PYE*_*:HA-PYE-GFP* plants, and found that the PYE-GFP signal was visible both in the cytoplasm and nucleus under Fe-sufficient conditions, and the PYE-GFP proteins accumulate in the nucleus under Fe-deficient conditions (Fig. 4B). Quantification of fluorescence revealed that the nucleus/cytoplasm ratio of GFP intensity under Fe-sufficient conditions (0.28 ± 0.13) was lower than that (0.96 ± 0.11) under Fe-deficient conditions (Fig. 4B).

**Fig. 5.**
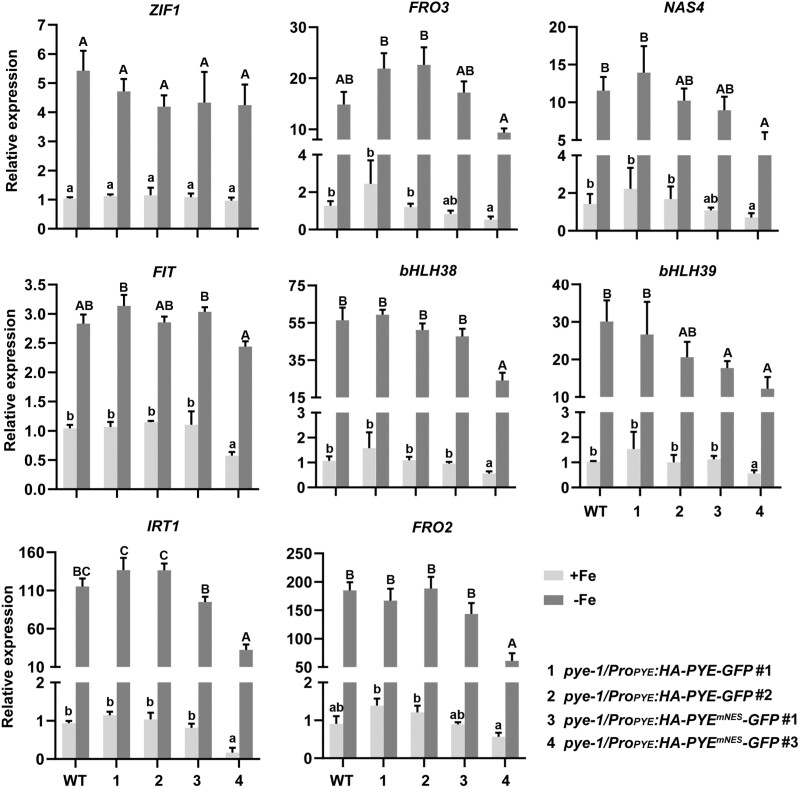
Expression of Fe deficiency-responsive genes, *ZIF1*, *FRO3*, *NAS4*, *FIT*, *bHLH38*, *bHLH39*, *IRT1* and *FRO2*, in *pye-1/Pro*_*PYE*_*:HA-PYE-GFP* and *pye-1/Pro*_*PYE*_*:HA-PYE*^*mNES*^*-GFP* plants. Plants were grown on +Fe medium for 4 d and then transferred to +Fe or –Fe medium for 3 d. RNA from root tissues was used for RT–qPCR. The expression levels were normalized to *ACT2* and *PP2A*. Data indicate means ±SD. Different letters (lower case for +Fe, and upper case for –Fe) above each bar indicate statistically significant differences as determined by one-way ANOVA followed by Tukey’s multiple comparison test (*P*<0.05).

A nuclear export signal (NES) consists of regularly spaced hydrophobic residues with several kinds of consensus patterns, and facilitates protein nuclear export (Xu *et al.*, 2015). We employed the NetNES tool (la Cour *et al.*, 2004) to predict potential NES sites of PYE, and found that PYE contains a NES site in its N-terminus (Fig. 3A). To clarify whether the NES has an impact on PYE sub-cellular localization, we generated the PYE^mNES^-GFP (a PYE version containing a mutated NES) construct. The transient expression assays in tobacco leaves indicated that PYE^mNES^-GFP is predominantly localized in the nucleus (Fig. 4A). To investigate whether the change of sub-cellular location of PYE would affect its function, we performed complementation assays. The *Pro*_*PYE*_*:PYE*^*mNES*^*-GFP* construct was introduced into the *pye-1* mutant plants (Supplementary Fig. S4B). We analysed 25 *pye-1*/*Pro*_*PYE*_*:PYE-GFP* and 32 *pye-1*/*Pro*_*PYE*_*:PYE*^*mEAR*^*-GFP* transformants, and found that all of them rescued the *pye-1* mutant (Figs 3C, 4C). In contrast, out of 30 *Pro*_*PYE*_*:PYE*^*mNES*^*-GFP* transformants, 21 transformants developed as well as the wild type, and nine transformants displayed the Fe-deficiency phenotypes as observed in the *pye-1*/*Pro*_*PYE*_*:PYE*^*mNES*^*-GFP#3* (Fig. 4D). The expression of *PYE* was comparable between *pye-1*/*Pro*_*PYE*_*:PYE-GFP#2* and *pye-1*/*Pro*_*PYE*_*:PYE*^*mNES*^*-GFP#3* (Supplementary Fig. S4B). We then examined the expression of some Fe deficiency-responsive genes, and found that their expression was lower in the *pye-1/Pro*_*PYE*_*:PYE*^*mNES*^*-GFP#3* than in the *pye-1*/*Pro*_*PYE*_*:PYE-GFP#2* (Fig. 5). These findings suggest that the mutation of NES causes constitutive nuclear localization and a stronger repressive function of PYE .

### bHLH104, bHLH105, and bHLH115 promote the nuclear localization of PYE

**Fig. 6.**
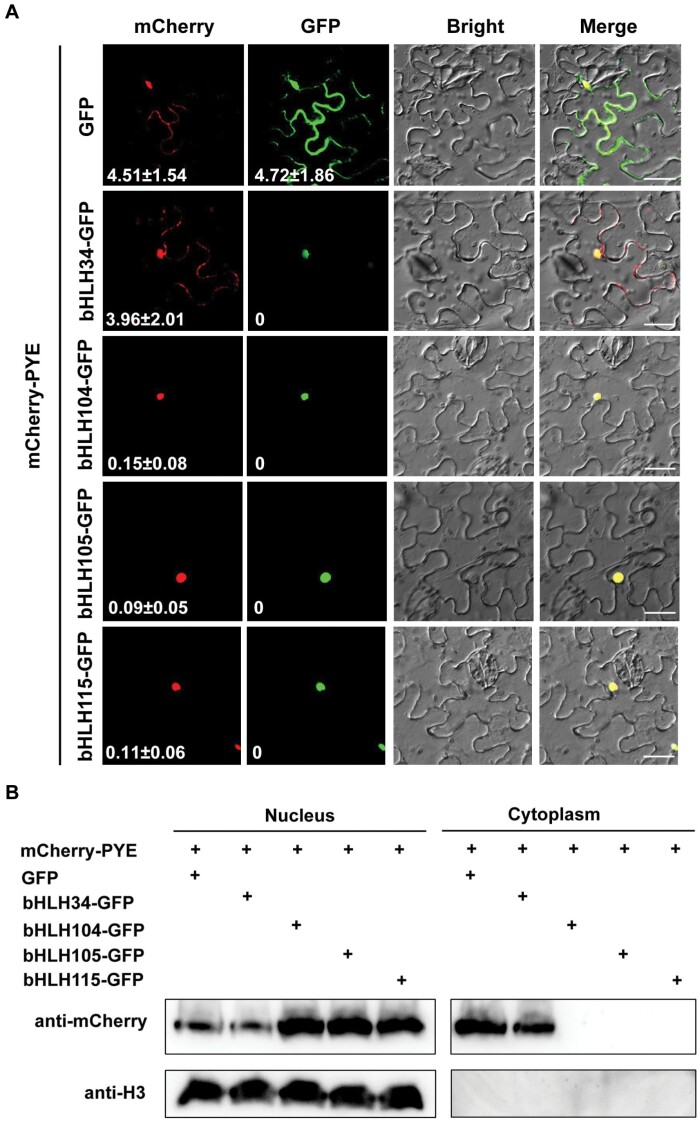
bHLH104, bHLH105, and bHLH115 promote the nuclear accumulation of PYE. (A) Sub-cellular localization of PYE. The mCherry-PYE was co-transformed with GFP, bHLH34-GFP, bHLH104-GFP, bHLH105-GFP, or bHLH115-GFP in tobacco leaves. After incubation in the dark for 2 d, GFP and mCherry signals were visualized under a confocal microscope. The numbers indicate the cytoplasm-to-nucleus ratio of fluorescence. The data represent means ±SD (*n*=10). Scale bars=20 μm. (B) Immunoblotting analysis of PYE protein in the cytosolic and nuclear fractions. Tobacco leaves from (A) were used for protein extraction and immunoblotting. Anti-mCherry and anti-Histone3 antibodies were used for immunoblot analysis.

Having confirmed that the NES is required for the cytoplasmic localization of PYE, we investigated what facilitates the nuclear accumulation of PYE. It was reported that PYE physically interacts with three out of the four bHLH IVc proteins, bHLH104, bHLH105, and bHLH115, which are predominantly localized in the nucleus (Long *et al.*, 2010; Selote *et al.*, 2015; Lei *et al.*, 2020). We questioned whether these bHLH IVc proteins contribute to the nuclear localization of PYE. The four GFP-tagged bHLH IVc proteins were individually co-expressed with mCherry-PYE. We found that mCherry-PYE was mainly localized in the nucleus in the presence of bHLH104-GFP, bHLH105-GFP, or bHLH115-GFP (Fig. 6A). In contrast, the presence of GFP or bHLH34-GFP did not affect the sub-cellular localization of mCherry-PYE. To further confirm that the sub-cellular localization of PYE is affected by its interaction partners, we performed immunoblotting analysis (Fig. 6B; Supplementary Fig. S5). When co-expressed with GFP or bHLH34-GFP, mCherry-PYE was detected both in the nucleus and cytoplasm. In contrast, mCherry-PYE was only detected in the nucleus with co-expression of bHLH104-GFP, bHLH105-GFP, or bHLH115-GFP. Therefore, we conclude that the PYE-interacting bHLH IVc proteins promote the nuclear localization of PYE.

### PYE represses the transcription activation ability of its interacting bHLH IVc members

**Fig. 7.**
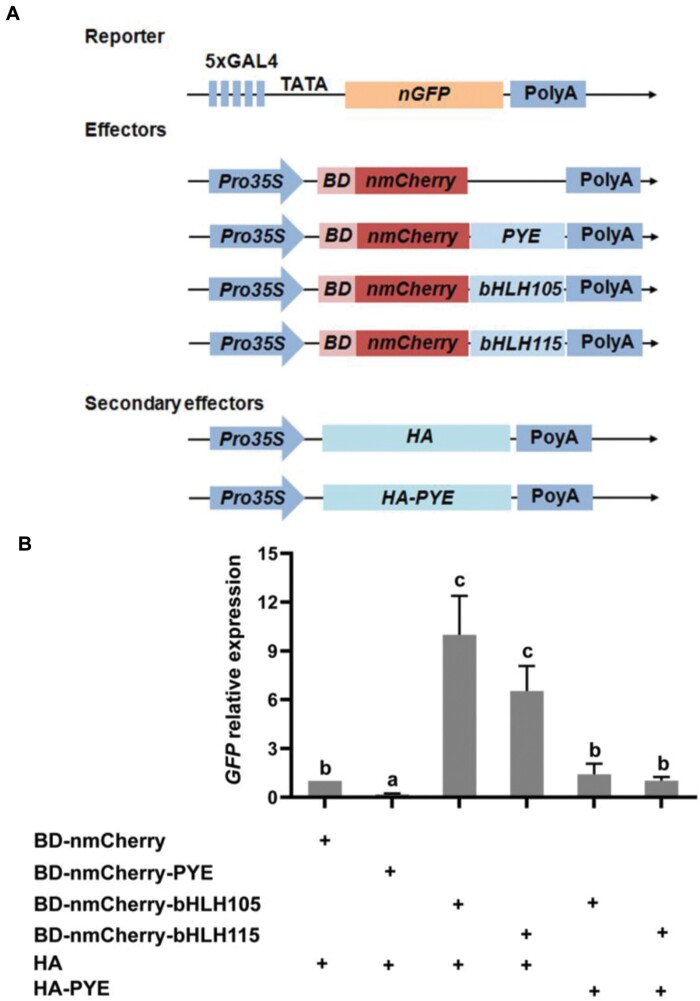
PYE represses the transcriptional activation ability of bHLH IVc. (A) The schematic diagram shows the constructs used in the transient expression assays. In the reporter, the minimal CaMV 35S promoter with five repeats of the GAL4 binding sequence drives the nuclear localized GFP (nGFP). In the effectors, the 35S promoter drives the fused genes in which the GAL4 DNA binding domain (BD), a target gene (PYE, bHLH105 or bHLH115) and the nuclear localized mCherry (nmCherry) were fused sequentially. HA and HA-PYE were used as the secondary effectors. (B) PYE represses transactivation of bHLH IVc. *Agrobacterium* cells with the reporter, the effector, or the secondary effector, were mixed and infiltrated into 3-week-old *Nicotiana benthamiana* leaves, and then kept in the dark for 48 h. The infiltrated leaves were harvested for RNA extraction and qRT–PCR. The abundance of *GFP* was normalized to that of *NPTII*. The value with the empty vector as an effector was set to 1. Each bar represents the mean ±SD of three independent experiments. Different letters above each bar indicate statistically significant differences as determined by one-way ANOVA followed by Tukey’s multiple comparison test (*P*<0.05).

Given that PYE negatively regulates the expression of bHLH Ib genes, we further questioned if PYE can antagonize the positive regulation function of bHLH IVc. Given the physical interaction of PYE with three bHLH IVc members (Long *et al.*, 2010; Selote *et al.*, 2015), we directly tested whether PYE inhibits their transcription activation by direct protein-protein interaction. We employed the GAL4-based effector-reporter system (Fig. 7A). Considering the similar molecular functions of bHLH IVc proteins, bHLH105 and bHLH115 were used as the representatives for further analysis. In the reporter, nGFP was driven by the minimal CaMV 35S promoter with five repeats of the GAL4 binding sequence. In the effectors, PYE, bHLH105, bHLH115 were fused with a nuclear localized mCherry (nmCherry) and the GAL4 DNA binding domain (BD), and driven by the 35S promoter. HA and HA-PYE were used as the secondary effectors. Consistent with the fact that bHLH IVc proteins are transcriptional activators and PYE is a repressor, the chimeric BD-nmCherry-bHLH105 and BD-nmCherry-bHLH115 activated the expression of *GFP* whereas BD-nmCherry-PYE inhibited its expression. Compared with the control (HA), the co-expression of HA-PYE with BD-nmCherry-bHLH105 or BD-nmCherry-bHLH115 significantly (*P*<0.05) reduced the expression of *GFP* (Fig. 7B). Taken together, these data suggest that PYE antagonizes the transcriptional activation ability of its bHLH IVc partners.

### PYE directly regulates its own expression and physically interacts with itself

**Fig. 8.**
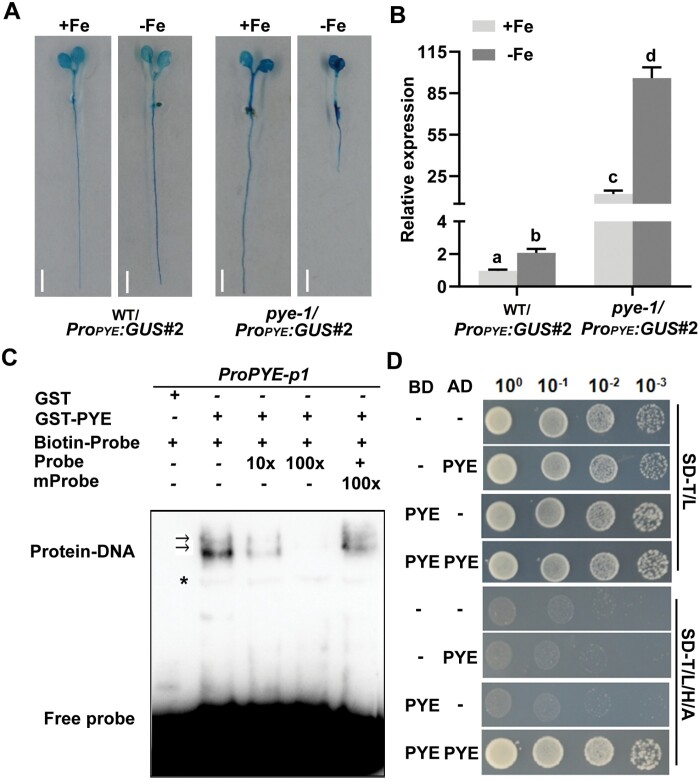
PYE directly regulates its own expression and physically interacts with itself. (A) GUS staining. One-week-old seedlings grown on +Fe or –Fe medium. Whole seedlings were subjected to GUS staining. Scale bars=5 mm. (B) Quantification of expression of *GUS*. Four-day-old plants grown on +Fe were transferred to +Fe or –Fe medium for 3 d, and root samples were used for RT–qPCR. Data indicate means ±SD. Different letters above each bar indicate statistically significant differences as determined by one-way ANOVA followed by Tukey’s multiple comparison test (*P*<0.05). (C) EMSA showing that PYE directly binds the *PYE* promoter. Either GST-PYE or GST was incubated with the biotin-labelled probes. Biotin-Probe, biotin-labelled probe; Probe, unlabelled probe; mProbe, unlabelled probe with mutated E-box. Biotin probe incubated with GST served as the negative control. Arrows indicate the GST-PYE/DNA complex. An asterisk indicates the non-specific band. (D) Yeast two-hybrid assays. Yeast co-transformed with different BD and AD plasmid combinations was spotted in parallel in a 10-fold dilution series. Growth on selective plates lacking Leu/Trp (SD–W/L) or Trp/Leu/His/Ade (SD–W/L/H/A).

bHLH Ib genes and *PYE* are directly regulated by bHLH IVc members and bHLH121 (Zhang *et al.*, 2015; Selote *et al.*, 2015; Li *et al.*, 2016; Liang *et al.*, 2017; Kim *et al.*, 2019; Gao *et al.*, 2020; Lei *et al.*, 2020). Having confirmed that PYE negatively regulates bHLH Ib gene expression, we wanted to know if PYE also negatively regulates its own expression. The *PYE* promoter was used to drive the GUS reporter, and then this construct (*Pro*_*PYE*_*:GUS*) was introduced into the wild type plants. GUS staining indicated that the *PYE* promoter is active in both the root and cotyledon (Fig. 8A). The transgenic line WT/*Pro*_*PYE*_*:GUS#2* was crossed with the *pye-1*, and the homozygous *pye-1*/*Pro*_*PYE*_*:GUS#2* line was identified. GUS staining analysis indicated that the *PYE* promoter activity is stronger in the *pye-1* than in the wild type (Fig. 8B). To further quantify the activity of the *PYE* promoter, we determined the expression levels of the *GUS* gene. We found that the transcript abundance of *GUS* was higher in *pye-1* than in wild type, irrespective of Fe status (Fig. 8B), suggesting that *PYE* promoter activity is negatively regulated by the PYE protein.

Chromatin immunoprecipitation (ChIP) assays in a transgenic line expressing the *Pro*_*PYE*_*:gPYE:GFP* (in *pye-1*) using an anti-GFP antibody found that the *PYE* promoters were enriched (Tissot *et al.*, 2019). Given that the same promoter regions were also enriched by bHLH105 protein (Tissot *et al.*, 2019), it is possible that PYE indirectly binds to its own promoter via the PYE-bHLH105 complex. To test whether the PYE protein is able to bind to its own promoter, EMSAs were performed, showing that PYE specifically binds to the *PYE* promoter (Fig. 8C).

Considering that bHLH proteins function as dimers, we queried whether PYE can form homodimers. The yeast two-hybrid assays indicated that homodimerization occurs in PYE proteins (Fig. 8D). Taken together, these results show that PYE physically interacts with itself to form dimers and represses its own expression by directly binding to its own promoter.

## Discussion

Fe deficiency is harmful to growth and development of plants. Plants can sense Fe deficiency conditions and modulate the expression of Fe deficiency-responsive genes in order to maintain Fe homeostasis. Many transcription factors, especially bHLH proteins, play pivotal regulatory roles in the Fe deficiency response signalling pathway (Gao and Dubos, 2021; Riaz and Guerinot, 2021; Liang, 2022). PYE was characterized as a negative regulator of the Fe homeostasis associated genes, *ZIF1*, *FRO3*, and *NAS4*, however, the loss-of-function of *PYE* causes enhanced sensitivity to Fe deficiency (Long *et al.*, 2010). The mechanism by which PYE regulates Fe homeostasis remains unclear.

**Fig. 9.**
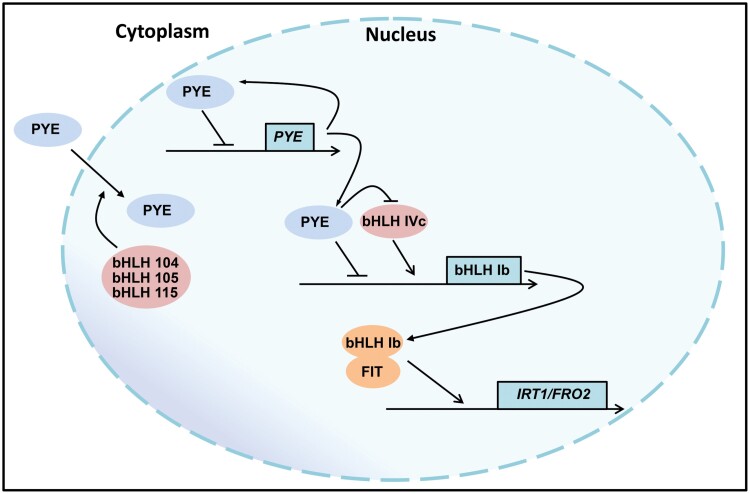
A working model of PYE function. Three out of four bHLH IVc proteins, bHLH104/105/115, interact with, and promote PYE accumulation in the nucleus. On the one hand, PYE negatively regulates the expression of bHLH Ib genes by direct association with their promoters; on the other hand, PYE indirectly regulates bHLH Ib by inhibiting the transactivity of bHLH IVc to bHLH Ib genes.PYE also negatively regulates its own transcription. bHLH Ib and FIT form dimers to initiate the expression of Fe uptake genes *IRT1* and *FRO2*. An arrow indicates a positive effect and a blunt arrow indicates a negative effect.

PYE was thought to regulate Fe homeostasis in an FIT-independent manner, since loss of function of *PYE* does not change the expression of FIT target genes *IRT1* and *FRO2* under Fe-deficient conditions (Long *et al.*, 2010; Riaz and Guerinot, 2021). Here, we provide evidence that *PYE* overexpression represses bHLH Ib genes, resulting in the down-regulation of FIT-dependent Fe uptake genes. FIT is a master regulator of Fe uptake systems because it is crucial for the up-regulation of Fe uptake genes, such as *IRT1* and *FRO2*, under Fe-deficient conditions (Colangelo and Guerinot, 2004; Jakoby *et al.*, 2004; Yuan *et al.*, 2008; Schwarz and Bauer, 2020). We found that the overexpression of *PYE* causes the down-regulation of *IRT1* and *FRO2*, and enhanced sensitivity to Fe deficiency (Fig. 1). We further confirmed that PYE directly represses the expression of bHLH Ib genes, *bHLH38* and *bHLH39*, by association with their promoters (Fig. 2). It is well known that the bHLH Ib proteins interact with FIT as heterodimers to regulate Fe uptake genes (Fig. 9; Yuan *et al.*, 2008; Wang *et al*, 2013). We propose that the significant reduction in expression of bHLH Ib genes is the reason why *IRT1* and *FRO2* are down-regulated in the *PYEox* plants. Although PYE is a negative regulator of bHLH Ib, *pye-1* mutants are also sensitive to Fe deficiency (Fig. 1). It has been reported that PYE is required for photoprotection under Fe-deficient conditions and during both low light and Fe deficiency, *pye-1* mutant develops as well as wild type (Akmakjian *et al.*, 2021). Thus, the defect in photoprotection is the reason why *pye-1* is sensitive to Fe deficiency. Although bHLH Ib expression increases in *pye-1* under Fe-deficient conditions, *FIT* expression does not (Fig. 1C), which does not further result in the increase of FIT-bHLH Ib dimers. Thus, it is reasonable that the expression of *IRT1* and *FRO2* is not up-regulated in *pye-1*. We also noted that the Fe reductase activity decreased in *pye-1* with the expression of *FRO2* unchanged, consistent with the report by Long *et al.*, (2010). It is possible that PYE regulates the protein abundance of FRO2. Rice OsIRO3 and OsIRO2 are the counterparts of PYE and bHLH Ib, respectively (Liang, 2022). Similarly, OsIRO3 also negatively regulates *OsIRO2* by directly binding to its promoter (C. Li *et al.*, 2022). It implies that different plants employ a similar regulation to maintain Fe homeostasis.

Many negative regulators exert their repression function by recruiting transcriptional co-repressors. A recent study revealed that bHLH11, a homolog of PYE, functions as a repressor since its EAR motif can interact with the transcriptional co-repressors, TPL/TPRs. Due to the existence of an EAR motif in PYE, it was thought that PYE may recruit TPL/TPRs to inhibit its target gene transcription. However, our data negate this hypothesis. PYE, bHLH11, and bHLH121 belong to the bHLH IVb sub-group. It has been confirmed that bHLH11 contains two EAR motifs which contribute to its negative function by recruiting TPL/TPRs (Y. Li *et al.*, 2022). Although bHLH121 is the closest homolog of bHLH11, it is not a negative regulator of Fe homeostasis (Kim *et al.*, 2019; Gao *et al.*, 2020; Lei *et al.*, 2020). Thus, these three members have evolved different functions. Further exploring which factors contribute to the negative function of PYE will enhance our understanding of the Fe deficiency response signalling.

The balance of positive regulators and negative regulators is crucial for the maintenance of Fe homeostasis. In contrast to the slight response of *FIT* transcription to Fe deficiency, the response to bHLH Ib genes is intensive. Due to the interdependence between bHLH Ib and FIT, modulating the expression of bHLH Ib genes is sufficient to control the FIT-dependent Fe uptake genes. As the major positive regulators of the Fe deficiency response, the bHLH IVc sub-group proteins, bHLH34, bHLH104, bHLH105, and bHLH115, directly activate bHLH Ib genes (Fig. 9). bHLH121, one member of the bHLH IVb sub-group, is also required for the up-regulation of bHLH Ib genes, and bHLH121 directly binds to their promoters (Kim *et al.*, 2019; Gao *et al*, 2020; Lei *et al.*, 2020). In contrast, bHLH11 is a negative regulator of bHLH Ib genes, since it inhibits the transcription activation activity of bHLH IVc towards bHLH Ib genes (Y. Li *et al.*, 2022). Here, we suggest that PYE directly represses bHLH Ib genes by binding to their promoters. We noted that PYE, bHLH121 and bHLH IVc proteins bind to the same promoter regions of bHLH Ib genes (Fig. 2C; Zhang *et al.*, 2015; Kim *et al.*, 2019; Tissot *et al.*, 2019; Gao *et al.*, 2020; Lei *et al.*, 2020). Thus, it is likely that they compete with each other for binding to the promoters. However, PYE may indirectly repress bHLH Ib genes since it represses the transcription activation ability of bHLH105 and bHLH115 (Fig. 7B). Therefore, bHLH IVc and PYE antagonistically regulate the expression of bHLH Ib genes (Fig. 9). The reciprocal antagonistic regulations of these transcription factors enable plants to modulate the expression of bHLH Ib genes, finally fine-tuning Fe uptake. In addition to bHLH Ib genes, *PYE* must also be maintained at an appropriate level, since the non-expression or over-expression of *PYE* causes the damage in the growth of plants. We further show evidence that PYE directly represses its own expression. It is worth mentioning that bHLH IVc proteins directly activate the expression of *PYE* (Zhang *et al.*, 2015; Liang *et al.*, 2017). Therefore, both bHLH IVc and PYE determine the transcript abundance of *PYE*.

The nuclear localization of transcription factors is required for them to exert regulatory functions since they need to bind to target promoters in nuclei. It has been reported that the other two members of bHLH IVb sub-group, bHLH11 and bHLH121, localize in the cytoplasm and nucleus, and bHLH IVc proteins facilitate their accumulation in the nucleus (Lei *et al.*, 2020; Y. Li *et al.*, 2022). Although PYE was reported as a nuclear protein (Long *et al.*, 2010; Selote *et al.*, 2015), our evidence supports that PYE is localized both in the cytoplasm and nucleus. Further investigation is required to explain this difference. It is known that bHLH IVc proteins positively regulate the expression of bHLH Ib genes. Here, we reveal that PYE negatively regulates them. Thus, PYE and bHLH IVc antagonistically regulate Fe homeostasis (Fig. 9). The bHLH IVc-dependent nuclear accumulation of PYE (Fig. 6) is crucial for the maintenance of Fe homeostasis, because when nuclear localized *PYE*^*mNES*^ was driven by the native promoter in *pye-1*, 30% of transformants displayed Fe deficiency phenotypes. We noted that the transgenic lines displaying Fe deficiency phenotypes had higher expression level of *PYE* than those displaying wild type phenotypes (Supplementary Fig. S4B). It is possible that the amount of nuclear PYE is controlled by the three bHLH IVc proteins, and the nuclear PYE protein balances bHLH IVc function. When the nuclear input of PYE is out of the control of bHLH IVc (like in PYE^mNES^ overexpressing lines), the elevated nuclear PYE strongly inhibits bHLH Ib, resulting in reduced Fe uptake and enhanced Fe deficiency. Thus, the conditional nuclear localization of PYE is required for Fe homeostasis. Interestingly, PYE was found to move between root cells, and tissue-specific misexpression of PYE protein exacerbates the *pye-1* phenotypes (Muhammad *et al.*, 2022). Therefore, Fe-dependent sub-cellular and tissular localization of PYE protein is essential for the maintenance of Fe homeostasis. Under Fe-deficient conditions, the expression of *PYE* is induced, which means that plants need its inhibitory function to avoid the excessive induction of Fe homeostasis-associated genes. Indeed, the loss of this ‘brake’ reduces plants ability to survive Fe-deficient conditions, as shown in the *pye-1* mutant (Long *et al.*, 2010). In addition to bHLH IVb proteins, bHLH39 proteins are also preferentially expressed in the cytoplasm, and they exclusively stay in the nucleus in the presence of FIT (Trofimov *et al.*, 2019). It is still unknown if their cytoplasm localization is required for their functions. In rice, the bHLH Ib protein, OsIRO2, also localizes in the cytoplasm, and its partner OsFIT facilitates its accumulation in the nucleus (Liang *et al.*, 2020; Wang *et al.*, 2020). One of the rice bHLH IVb proteins, OsIRO3, was reported to be localized in the nucleus (Zheng *et al.*, 2010), and the sub-cellular localization of the other members has not been reported. Further investigation is required to clarify whether the cytoplasm retention phenomenon of bHLH IVb proteins universally exists across different plant species and is crucial for Fe homeostasis.

## Supplementary data

The following supplementary data are available at *JXB* online. 

Fig. S1. Relative expression levels of *PYE* in *PYEox* plants.

Fig. S2. Immunoblotting analysis of the purified PYE recombinant protein.

Fig. S3. The EAR motif of PYE is not required for its repression function.

Fig. S4. The NES is required for PYE functions.

Fig. S5. Immunoblotting analysis of bHLH IVc proteins.

Table S1. Primers used in this study.

erad057_suppl_Supplementary_Data

## Data Availability

The data supporting the findings of this study are available from the corresponding author (GL) upon request.
