# Supplementary material for: The transcription factor POPEYE negatively regulates the expression of bHLH Ib genes to maintain iron homeostasis
Source: J Exp Bot. 2023 Feb 14;74(8):2754–67. doi: 10.1093/jxb/erad057 (PMC10797486; doi:10.1093/jxb/erad057)
Supplement: erad057_suppl_Supplementary_Data [file erad057_suppl_supplementary_data.pdf]

The transcription factor POPEYE negatively regulates the expression of bHLH Ib genes for Fe homeostasis

Meng Na Pu[a, b], and Gang Liang[a, b,*]

[a]CAS Key Laboratory of Tropical Plant Resources and Sustainable Use, Xishuangbanna Tropical Botanical Garden, Kunming, Yunnan 650223, China

[b]The College of Life Sciences, University of Chinese Academy of Sciences, Beijing 100049, China

[*]Correspondence:

Gang Liang

Email: lianggang@xtbg.ac.cn

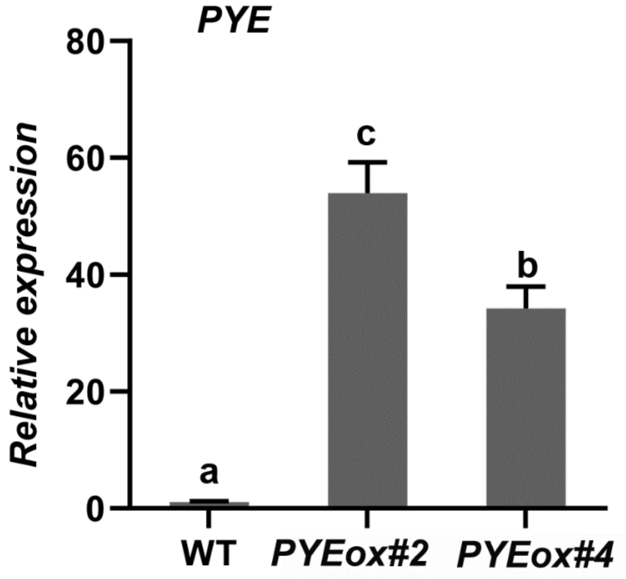

Supplemental Figure S1. Relative expression levels of *PYE* in *PYEox* plants. Roots from seedling grown on +Fe medium for 7 days were harvested for the extraction of RNA and qRT-PCR. The expression levels were normalized to *ACT2* and *PP2A*. The data represent means ± SD. The different letters above each bar indicate statistically significant differences as determined by one-way ANOVA followed by Tukey multiple comparison test ($P < 0.05$).

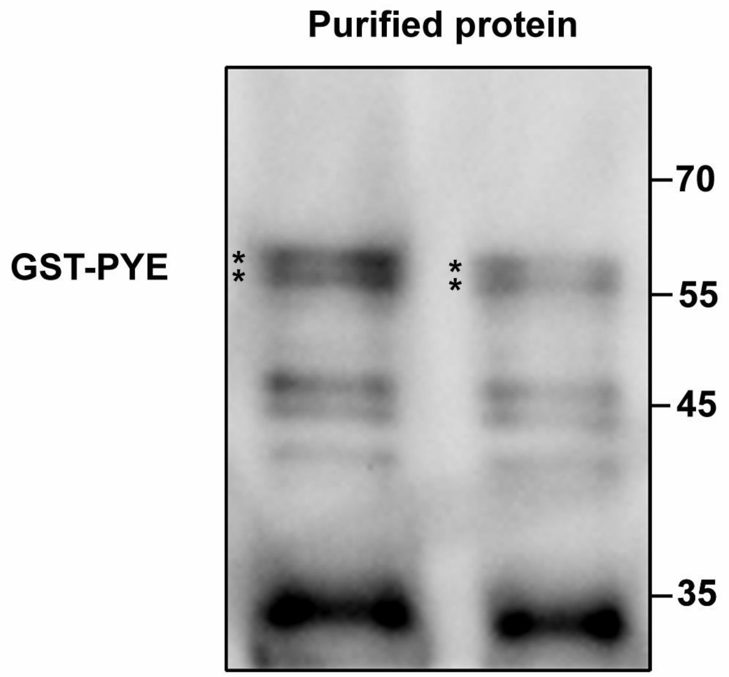

Supplemental Figure S2. Immunoblotting analysis of the purified PYE recombinant protein.

The recombinant GST-PYE protein was expressed in *E. coli*. Purified protein was used for immunoblotting analysis. Protein molecular weights (in kDa) are indicated.

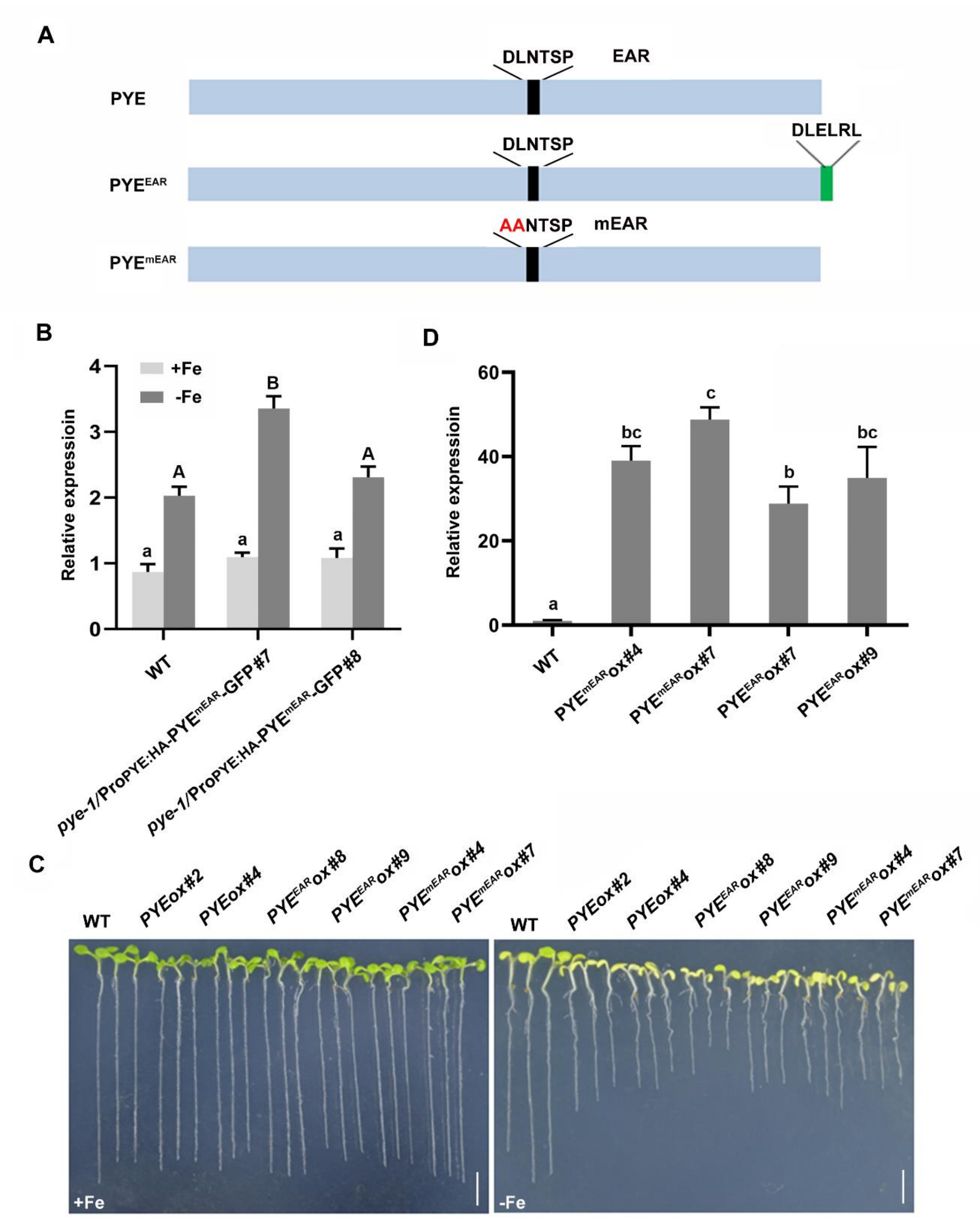

Supplemental Figure S3. The EAR motif of PYE is not required for its repression function.

(A) Schematic diagram of the various versions of PYE. The mutated amino acid is indicated in red. mEAR, the mutated EAR; PYE^{EAR}, the end of PYE was fused with an EAR domain.

(B) Relative expression levels of *PYE* in the *Pro$_{PYE}$:HA-PYE^{EAR}-GFP/pye-1*

plants. Four-day-old plants grown on +Fe were transferred to +Fe or –Fe medium for three days. Roots were harvested for the extraction of RNA and qRT-PCR. The expression levels are normalized to *ACT2* and *PP2A*. The data represent means ± SD. The different letters above each bar indicate statistically significant differences as determined by one-way ANOVA followed by Tukey multiple comparison test (*P* < 0.05).

(C) Phenotypes of various transgenic plants. One-week-old seedlings grown on +Fe or –Fe medium. Scale bars=4 mm.

(D) Relative expression levels of *PYE* in the PYE^{mEAR} and PYE^{EAR} plants. Roots of seven-day-old plants grown on +Fe were harvested for the extraction of RNA and qRT-PCR. The expression levels are normalized to *ACT2* and *PP2A*. The data represent means ± SD. The different letters above each bar indicate statistically significant differences as determined by one-way ANOVA followed by Tukey multiple comparison test (*P* < 0.05).

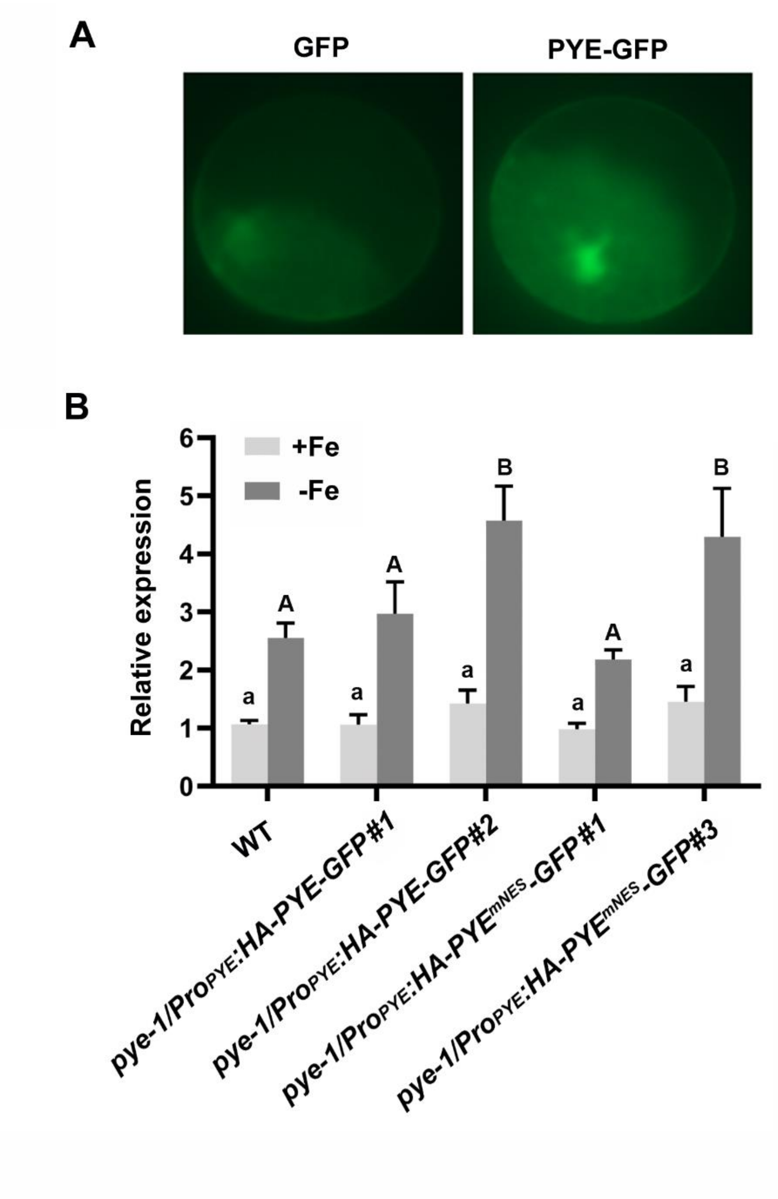

Supplemental Figure S4. The NES is required for PYE functions.

(A) Subcellular localization of PYE. GFP or PYE-GFP transformed into Arabidopsis mesophyll protoplasts. The GFP signal was visualized under a confocal microscope.

(B) Relative expression levels of *PYE* in various transgenic plants. Four-day-old plants grown on +Fe were transferred to +Fe or –Fe medium for three days. Roots were harvested for the extraction of RNA and qRT-PCR. The expression levels are normalized to *ACT2* and *PP2A*. The data represent means ± SD. The different letters above each bar indicate statistically significant differences as determined by one-way ANOVA followed by Tukey

multiple comparison test ($P < 0.05$).

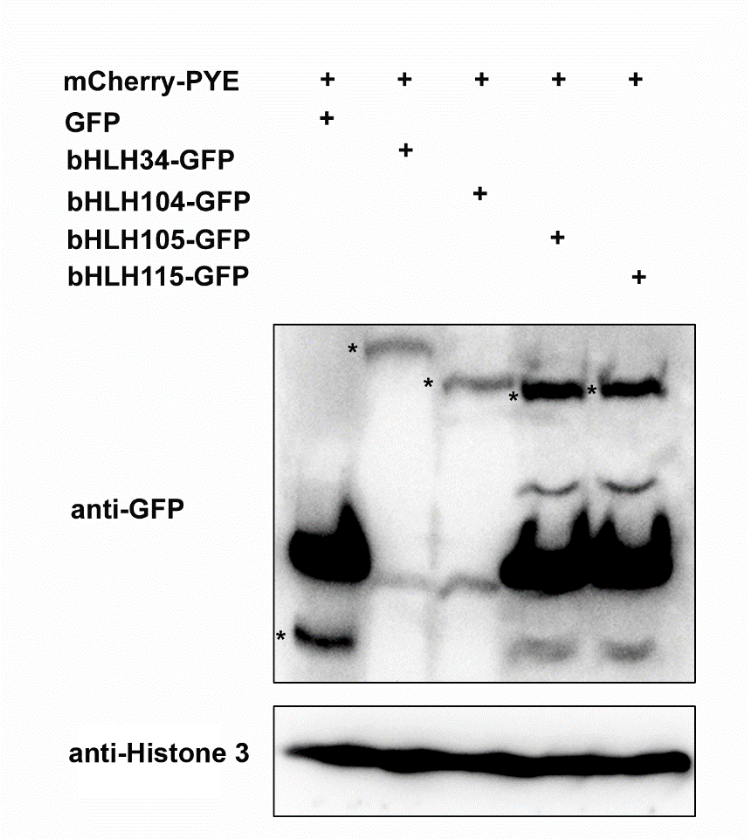

Supplemental Figure S5. Immunoblotting analysis of bHLH IVc proteins. Tobacco leaves in Figure 6 were harvested for extraction of total protein. Anti-GFP and anti-Histone3 antibodies were used for immunoblot analysis. Asterisks indicated the specific protein bands.

Supplemental Table S1. Primers used in this study.

PYE-F	ATAGGATCCATGGAGTACCCATACGACGTACCAGATTACGCTATGGTATCGAAAACTCCTTCTACAT	pOCA30	*PYEox*
PYE-R	ATAGTCGACTCATTCACTGGCTTTCAGCC		
PYE^{mEAR}-F	GTCGAAACCTGcCgcGAACACCTCTCCTGCACCCG	pOCA30	*PYE^{mEAR}ox*
PYE^{mEAR}-R	GAGGTGTTCgcGgCAGGTTTCGACTGATTCGCTCT		
PYE^{mNES}-F	gcTGAATTAGCCGATACTCTTGAAgcGAATCAACAGAACAGTGGGAAAG	pOCA30	*PYE^{mNES}ox*
PYE^{mNES}-R	CgcTTCAAGAGTATCGGCTAATTCAgcGAAAAGCTCATTCAAATGCTC		
PYE-F	ATAGGATCCATGGAGTACCCATACGACGTACCAGATTACGCTATGGTATCGAAAACTCCTTCTACAT	pOCA30	*PYE^{mEAR}ox*
PYE^{EAR}-R	ATAGTCGACTACAAACGGAGTTCGAGATCTTCACTGGCTTTCAGCCGCT		
pPYE-PYE-F	GAAAGAATTCGAGCTCGCCCGGGCGAACCGCAAAACTATATATAGTA	p28-GFP	*Pro$_{PYE}$:HA-PYE-GFP*
pPYE-PYE-R	CTGGTACGTCGTATGGGTACTCCATCTTTGCTTTTATTACAGAACAAGA	p28-GFP	*Pro$_{PYE}$:HA-PYE^{mEAR}-GFP*
PYE-HA-F	ATGGAGTACCCATACGACGTACCAGATTACGCTATGGTATCGAAAACTCCTTCTACAT	p28-GFP	*Pro$_{PYE}$:HA-PYE^{mNES}-GFP*
PYE-GFP-R	GCCCTTGCTCACCATGGTTCTAGAGTCACTGGCTTTCAGCCGCTC	p28-GFP	
pPYE-F	TTgtcgaCGAACCGCAAAACTATATATAGTA	p28-GUS	*Pro$_{PYE}$:GUS*
pPYE-R	AAggatcCTTTGCTTTTATTACAGAACAAGA		
GAD-PYE-F	TTTGAATTCATGGTATCGAAAACTCCTTCTACA	pGAD-T7	*pGAD-PYE*
GAD-PYE-R	TTTGGATCCTCATTCACTGGCTTTCAGCC		
